# Optimization of Interfacial Properties Improved the Stability and Activity of the Catalase Enzyme Immobilized on Plastic Nanobeads

**DOI:** 10.1021/acs.langmuir.4c01508

**Published:** 2024-07-27

**Authors:** Szilárd Sáringer, Gergő Terjéki, Árpád Varga, József Maléth, István Szilágyi

**Affiliations:** † MTA-SZTE Lendület Biocolloids Research Group, Interdisciplinary Excellence Center, Department of Physical Chemistry and Materials Science, University of Szeged, H-6720 Szeged, Hungary; ‡ MTA-SZTE Lendület Epithelial Cell Signaling and Secretion Research Group, Interdisciplinary Excellence Centre, University of Szeged, H-6720 Szeged, Hungary

## Abstract

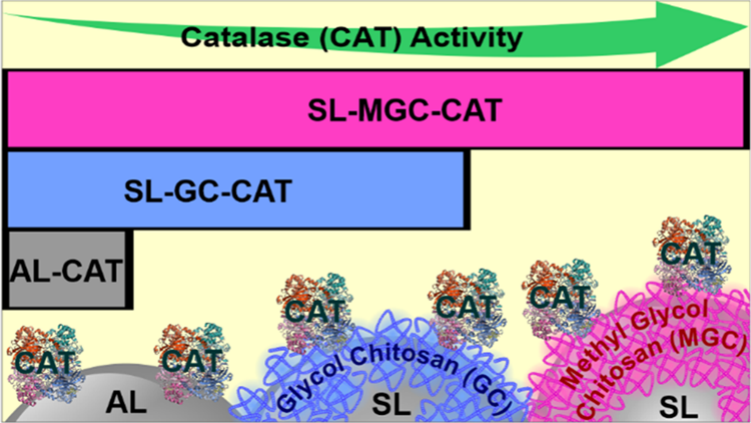

The immobilization of catalase (CAT), a crucial oxidoreductase enzyme involved in quenching reactive oxygen species, on colloids and nanoparticles presents a promising strategy to improve dispersion and storage stability while maintaining its activity. Here, the immobilization of CAT onto polymeric nanoparticles (positively (AL) or negatively (SL) charged) was implemented directly (AL) or via surface functionalization (SL) with water-soluble chitosan derivatives (glycol chitosan (GC) and methyl glycol chitosan (MGC)). The interfacial properties were optimized to obtain highly stable AL-CAT, SL-GC-CAT, and SL-MGC-CAT dispersions, and confocal microscopy confirmed the presence of CAT in the composites. Assessment of hydrogen peroxide decomposition ability revealed that applying chitosan derivatives in the immobilization process not only enhanced colloidal stability but also augmented the activity and reusability of CAT. In particular, the use of MGC has led to significant advances, indicating its potential for industrial and biomedical applications. Overall, the findings highlight the advantages of using chitosan derivatives in CAT immobilization processes to maintain the stability and activity of the enzyme as well as provide important data for the development of processable enzyme-based nanoparticle systems to combat reactive oxygen species.

## Introduction

Catalase (CAT) enzyme plays a pivotal role in the intricate web of biochemical processes that sustain life.^1,2^ Its essential function in maintaining cellular homeostasis and protecting organisms from oxidative stress has attracted researchers for decades.^3,4^ CAT possesses a remarkable ability to decompose hydrogen peroxide (H_2_O_2_), a reactive oxygen species that poses a significant threat to cellular integrity when left unchecked.^5^ Remarkably, CAT has the highest turnover numbers of all enzymes, converting millions of H_2_O_2_ molecules to water and oxygen per second,^6^ which is the main reason for its industrial applications. For instance, the elimination of this harmful substrate usually leads to improved product quality and extended shelf life of food^7^ and textile^8^ products. In addition, CAT is useful in various technological and biomedical applications,^9−11^ including eco-friendly waste treatment processes, prevention of coal oxidation, and antioxidant therapies.

Like most enzymes, CAT is highly sensitive to changes in environmental conditions such as temperature, pH, and the presence of inhibitors or activators.^12^ Even slight variations in these parameters can significantly impact enzyme activity and stability, making it crucial to formulate these proteins for wider application fields. In this way, the immobilization of enzymes on solid supports is a promising formulation strategy,^13−17^ as it provides improved enzyme stability, better enzyme reusability, and allows easy separation from the reaction mixture, which are important factors in the industrial use of enzymes.^18^ Immobilization on solid supports also offers enhanced or maintained enzyme activity, and thus, it has become a valuable strategy in biocatalysis. Frequently used strategies for enzyme attachment to solid supports include immobilization via physical adsorption and covalent linkage.^13,19−22^ Physical immobilization is often preferred over attachment by primary (covalent) chemical bonds due to the milder reaction conditions and simpler protocols.^23^ Nevertheless, the latter method is appropriate to prevent enzyme leakage, which is one of the major limitations of immobilization via physical adsorption.

Nanoparticles are excellent candidates as support matrices, as they provide high a surface area, efficient enzyme loading, and improved catalytic activity.^24−27^ Moreover, the unique physicochemical properties of nanoparticles, such as size, shape, and surface chemistry, can be tailored to optimize the immobilization process and enhance the stability of immobilized enzymes. For instance, several studies have investigated the immobilization of CAT on various types of nanoparticles and their composites. Accordingly, magnetic,^28^ silica,^29^ clay,^30^ and noble metal^10^ nanoparticles as well as hybrid nanocomposites^31−33^ or polymeric materials^34^ have been used as supports for CAT. However, it is important to note that surface properties play a crucial role in achieving successful enzyme immobilization. Currently, to the best of our knowledge, there is a lack of systematic studies investigating the specific impact of interfacial characteristics on CAT activity upon immobilization.

Furthermore, as a consequence of the features of the solid–liquid interface, colloidal stability is a critical factor in nanoparticle-based enzyme immobilization.^35−37^ It refers to the ability of nanoparticles to maintain their dispersed state and resist aggregation and subsequent phase separation. Such stability is of paramount importance for ensuring efficient function and long-term use of the immobilized enzyme system. In addition, optimizing colloidal stability allows for enhanced enzyme loading and improved catalytic activity. Moreover, stable nanoparticle systems exhibit improved resistance to harsh reaction conditions, such as changes in temperature and pH, thereby enhancing the overall stability and longevity of the immobilized enzyme.^25,26,38^

The application of polyelectrolytes is a self-evident way to tune the colloidal properties of enzymes immobilized on nanoparticulate supports. Among weak polyelectrolytes, chitosan, a natural polysaccharide derived from chitin, is a potential candidate, but it is insoluble in water due to its molecular structure.^39^ However, through chemical modifications, water-soluble chitosan derivatives have been developed, expanding their potential applications.^40^ Accordingly, these derivatives have shown promising pH-responsive antimicrobial, antioxidant, and wound healing properties, making them excellent building blocks in materials used in drug delivery,^41^ food processing,^42^ and packaging.^43^

In this study, the CAT enzyme was immobilized on bare and coated polymeric nanobeads using water-soluble chitosan derivatives, namely glycol chitosan (GC) and methyl glycol chitosan (MGC), as coating agents. The doses of both polyelectrolytes and enzymes were carefully optimized to ensure the high colloidal and functional stability of these composites. The colloid approach applied in CAT immobilization can be recommended for future industrial applications, wherever the goal is to develop H_2_O_2_ decomposing agents in heterogeneous systems.

## Experimental Section

### Materials

Styrene (99%) and 2,2′-azobis(2-methylpropionamidine) (AIBA) were purchased from Acros Organics. Potassium peroxodisulfate (KPS), hydrogen peroxide (H_2_O_2_), and sodium chloride (NaCl) were purchased from VWR. Catalase (CAT, 2–5 U/mg, dried) derived from bovine liver, methyl glycol chitosan (MGC), glucose oxidase, phosphate-buffered saline (PBS), glucose, and cysteamine hydrochloride were acquired from Sigma-Aldrich. Paraformaldehyde (PFA) and ammonium molybdate ((NH_4_)_6_Mo_7_O_24_·4H_2_O) were obtained from Alfa Aesar. Primary antibodies against CAT and the fluorophore-conjugated secondary antibody (donkey antimouse Alexa 647) were purchased from Thermo Fisher Scientific. Glycol chitosan (GC) was procured from MP Biomedicals. All chemicals used were of analytical grade and used as received. Ultrapure water (UPW) from an ADRONA B30 machine was used for sample preparation, and both water and salt solutions were filtered using 0.1 μm syringe filters (Millex). The measurements were conducted at 25 °C unless otherwise indicated.

### Synthesis of Polymer Nanoparticles

Polystyrene nanoparticles with different surface functionalities were prepared by stabilizer and emulsifier-free emulsion polymerization.^44,45^ The negatively charged sulfate latex (SL) particles and the positively charged amidine latex (AL) particles were prepared using the same radical polymerization procedure, except that the initiators were KPS and AIBA, respectively. First, 898 mL of ultrapure water was heated to 80 °C in a refluxed three-neck round-bottomed flask. The solvent was bubbled with N_2_ and stirred at 500 rpm for 15 min. Then, 2 g (0.2 wt %) of styrene monomer was added to the solution, and the emulsion was stirred for another 15 min under an inert atmosphere. Simultaneously, a 0.2 g initiator (KPS or AIBA) was dissolved in 100 mL of N_2_-bubbled UPW and added to the above emulsion. The solution was stirred (500 rpm) and refluxed for 24 h under an N_2_ atmosphere. Since the polymer concentration was low (0.1–0.2 g/L) in the mother liquid, it was necessary to concentrate the sample with solvent evaporation at 50 °C, and the volume was reduced to 100 mL. The resulting SL and AL dispersions were dialyzed against UPW for 3 days. The purification process was continued until the water conductivity was reached. The concentration of the final stock dispersion was 10 g/L.

### Design of the Composite Materials

**Scheme 1:**
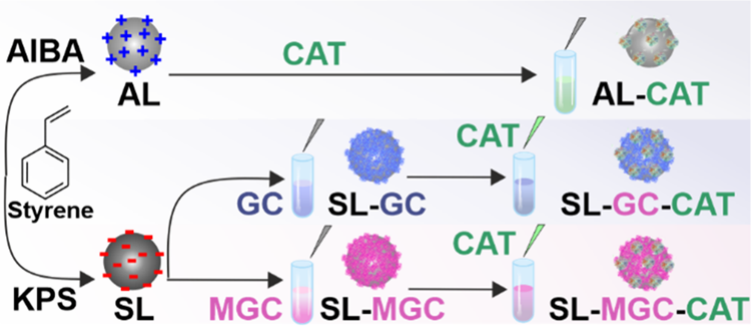
Schematic Illustration of the Preparation of the Composite Materials

The preparation of AL-CAT, SL-GC-CAT, and SL-MGC-CAT involved a sequential adsorption process,^30,37,46^ in which polyelectrolytes and enzymes were deposited onto oppositely charged latex particles via electrostatic forces. The optimal doses were determined using electrophoretic measurements, as discussed later. For instance, for AL-CAT, the process commenced by adding AL particles to a solution containing an appropriate amount of CAT at pH 7 and stirring for 1 h. Besides, the SL particles were dispersed in a solution comprising a predefined quantity of GC or MGC and stirred for 1 h. Subsequently, a calculated amount of CAT was introduced into the solution and stirred for an additional hour, resulting in the formation of SL-GC-CAT or SL-MGC-CAT composite dispersion. To adjust the high colloidal stability, the doses of polyelectrolytes and enzymes were 100 and 10 mg/g (relative to the mass of the particles), respectively, as discussed later. The preparation process is shown in Scheme 1.

### Dynamic Light Scattering

The hydrodynamic radius (*r*_h_) of the particles was measured by dynamic light scattering (DLS) experiments with a CGS-3 compact goniometer system (ALV, 35 mW He–Ne laser with a wavelength of 633 nm and a scattering angle of 90°). The correlation function was determined for 20 s, and the cumulant fit was used to obtain the decay rate, and subsequently, the translational diffusion coefficients, which were converted to *r*_h_ with the Stokes–Einstein equation.^47^ The aggregation rate coefficient during dimer formation was determined in a time-resolved mode by monitoring the change in the size. The relationship for the rate of change of the hydrodynamic radius (d*r*_h_/d*t*) as the experiment time (*t*) approaches zero (early stages of aggregation) is given as^48^
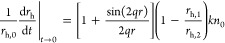
1In the above equation, *n*_0_ represents the initial particle number concentration (2.27 × 10^14^ 1/m^3^), *r*_h,0_ is the initial hydrodynamic radius, *q* is the magnitude of the scattering vector, *r* is the geometrical radius, and *r*_h,1_/*r*_h,2_ is the ratio of the hydrodynamic radii of the monomer and the dimer. The measurements were conducted for 25 min to ensure enough data points for linear fits of the *r*_h_ versus *t* data. The sample volume used finally was 2 mL. During polyelectrolyte or enzyme dose-dependent measurements, 0.2 mL of 60 mg/L bare or functionalized particle stock dispersion was added to 1.8 mL of a solution composed of a calculated amount of polyelectrolyte or enzyme stock, with an ionic strength of 1 mM. In the case of ionic strength-dependent measurements, the particles were added to a calculated amount of NaCl and polyelectrolyte solution. The samples were vortexed and immediately measured using DLS. The colloidal stability of the particles is expressed by calculating the stability ratio (*W*) as^49^
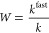
2The fast condition corresponds to diffusion-controlled particle aggregation achieved in 1 M NaCl solutions, at which electrostatic repulsion is screened.^50^ Stability ratio values close to unity indicate unstable dispersions, i.e., diffusion-controlled aggregation, where all particle collisions result in dimer formation. The mean error of the stability ratio data is within 10%. In salt-induced aggregation studies, the critical coagulation concentration (CCC) is calculated using the following equation^51^
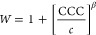
3In this equation, *c* refers to the molar salt concentration and β is obtained from the change in the stability ratios in the slow aggregation regime before the CCC as
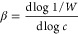
4

### Electrophoretic Light Scattering

The electrophoretic mobility (μ) of the particles was measured by electrophoresis using a phase analysis light scattering mode^52^ with a Litesizer 500 instrument (Anton Paar) equipped with a 40 mW semiconductor laser operating at a wavelength of 658 nm. The measurements were carried out in omega cuvettes (Anton Paar). The μ data were converted to zeta potentials (ζ) using Smoluchowski’s equation^53^
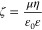
5where ε is the relative permittivity of water (78.5), ε_0_ is the permittivity of vacuum (8.9 × 10^–12^ F/m), and η is the viscosity of water (8.9 × 10^–4^ Pa·s at the respective temperature). The surface charge density at the slip plane (σ) was calculated from the ionic strength dependence of ζ using the Grahame equation^49^
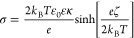
6Here, *e* is the elementary charge, *k*_B_ is the Boltzmann constant, *T* is the temperature, and κ is the inverse Debye length. For all measurements, 1 mL samples were prepared, following the same sample preparation method used in the DLS measurements, with the only difference being that the samples were allowed to rest for 2 h at room temperature before recording the μ data after 1 min equilibrating time in the device. To check reproducibility, the ζ values of the samples used for the DLS experiments were also measured. For pH-dependent measurements, SL, AL, and CAT aqueous samples were prepared at pH values of 3 and 11. The concentrations were 10 and 100 mg/L in the particle dispersion and enzyme solution, respectively. The acidic and basic dispersions were mixed in different ratios, and μ was determined after pH measurement with a WTW7310 benchtop pH meter. Thus, the particle or enzyme concentration and ionic strength (1 mM) were constant, and only the pH was varied in the samples.

### Transmission Electron Microscopy

The morphologies of the AL and SL particles were examined by transmission electron microscopy (TEM). The dispersions were dried on a copper-coated carbon mesh TEM grid followed by imaging with a TECNAI G2 20 X-TWIN instrument (FEI) at an accelerating voltage of 200 kV.

### Immunofluorescence Labeling and dSTORM Imaging

The immobilization of CAT in the AL-CAT and SL-GC-CAT composites was visualized by direct stochastic optical reconstruction microscopy (dSTORM).^54,55^ First, the particle dispersions were placed on a cover glass and incubated for 20 min to achieve adsorption. After 5 min of fixation with 4% PFA in PBS, specific binding sites were blocked using 10% BSA in PBS for 2 h at 37 °C. To mark the enzyme, an anti-CAT primary antibody was used (2 h incubation at room temperature), and thereafter, the samples were washed 3 times for 5 min with PBS. A fluorophore-conjugated (Alexa 647) secondary antibody was applied prior to another washing step (3 times 5 min). For dSTORM imaging, cover glasses were placed on cavity slides filled with a blinking buffer and sealed with two adhesive components. The blinking buffer contained 100 U glucose oxidase, 2000 U CAT, 55.6 mM glucose, and 100 mM cysteamine hydrochloride in a 1 mL final volume completed with sterile PBS. The dSTORM images were recorded using a Nanoimager S (Oxford Nanoimaging ONI Ltd.) microscope.

### CAT Activity

To assess the CAT activity of the native and immobilized enzymes, a standard spectrophotometric assay^56^ was used, with slight modifications. This probe is based on the formation of a yellow-colored product during the reaction between H_2_O_2_ and ammonium molybdate, with an absorbance maximum at a wavelength of 346 nm. To record the calibration curve, 1 mL of 37.5 mM ammonium molybdate was added to a 0.5 mL standard solution, in which the H_2_O_2_ concentration was between 1 and 15 mM, except for the blank sample, which did not contain any H_2_O_2_. The calibration curve showed linearity (RSQ 0.999) up to 7 mM H_2_O_2_ concentration (Figure S1); therefore, the activity measurements were performed at 6.7 mM. For CAT activity measurements, the total reaction mixture volume was 1.5 mL. First, 0.03–0.15 mL of bare or immobilized CAT stock solution was completed to 0.3 mL with UPW. Second, 0.2 mL of 50 mM H_2_O_2_ was added to the reaction mixture. Third, after 3 min reaction time, 1 mL of 37.5 mM ammonium molybdate was introduced to the samples, which were then centrifuged for 10 min at 10,000 rpm, and the absorbance of the supernatant was measured at 346 nm. Based on the measured absorbance values, the amount of decomposed H_2_O_2_ at different CAT concentrations was calculated. For short-term stability measurements, 8 mL of 50 mM H_2_O_2_ was added to 12 mL of enzyme solution with a CAT concentration of 0.2 mg/L. Subsequently, 0.5 mL of the solution was withdrawn at different times during a 2-h period, after centrifugation, the percentage of decomposed H_2_O_2_ was determined by adding 1 mL of a 37.5 mM ammonium molybdate solution to the supernatant. For the long-term stability tests, samples were prepared at a CAT concentration of 4 mg/L. During a 6-day period, 0.3 mL aliquot was withdrawn from the samples each day and mixed with 0.2 mL of 50 mM H_2_O_2_. After a reaction time of 3 min, 1 mL of 37.5 mM ammonium molybdate was added, and the absorbance was measured after centrifugation. In the reusability tests, 9.6 mL samples were prepared at a 2.5 mg/L CAT concentration. For 5 days, a calculated amount of 500 mM H_2_O_2_ was added every day to maintain a substrate concentration of 6.7 mM in the samples. Since the CAT dose was changed each day in the samples, the results were always normalized to the actual CAT concentration. The samples were taken out and treated in the same way as in the long-term stability test, and both assessments were performed at room temperature. Note that this method is appropriate for comparing the activities of bare and immobilized enzymes, without the need to perform detailed kinetic experiments, which can be applied to further investigate enzyme–particle interactions at the interface.

## Results and Discussion

### Characterization of Polystyrene Latex Nanoparticles

**Figure 1:**
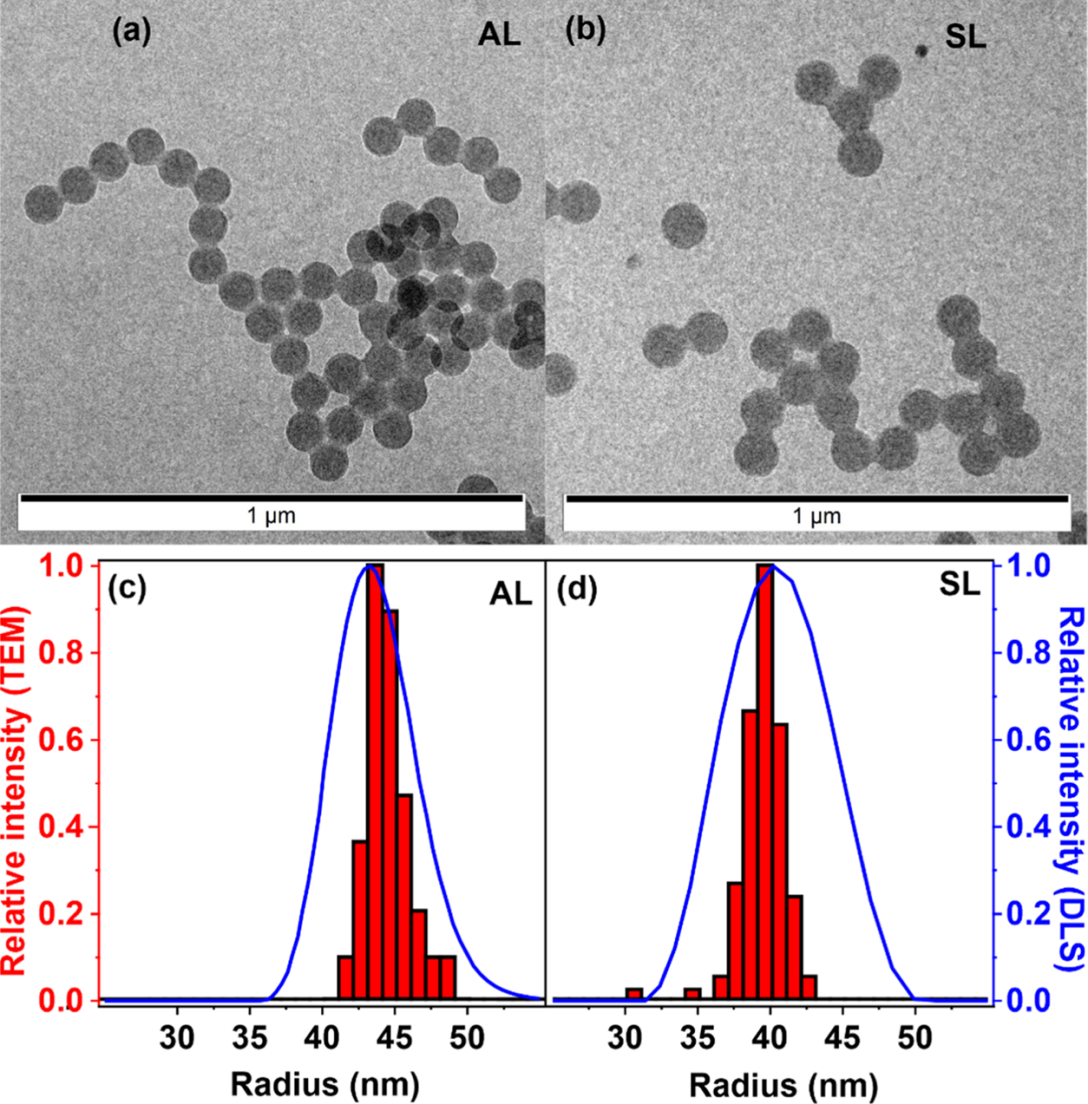
TEM images of AL (a) and SL (b) particles. Size distribution by TEM (bars, left axis) and DLS (lines, right axis) of AL (c) and SL (d) nanobeads.

Polystyrene-based nano- and colloidal particles (like AL and SL in the present work) are excellent candidates for enzyme immobilization in colloidal systems due to their controllable synthesis, narrow size distribution, and possible tuning of the surface charge properties. AL and SL beads were prepared by surfactant- and emulsifier-free emulsion polymerization with different initiators to obtain positively and negatively charged nanoparticles, respectively. The TEM images in Figures 1a,b and S2 reveal that both types of particles have regular spherical shapes and monodisperse size distributions.

From the TEM measurements, the average radii of the particles were 44.4 ± 1.5 and 39.3 ± 1.6 nm for the AL and SL, respectively. The DLS measurements (Figure 1c,d) did not yield a significant difference in size, as the hydrodynamic radii were 44.2 ± 0.8 and 43.9 ± 1.8 nm for the AL and SL, respectively. The size distribution was determined using both methods, supporting a monodisperse size distribution. As expected, DLS measurements show a somewhat wider size distribution since the number of particles detected in DLS is orders of magnitude greater than the ones counted in the TEM images and, hence, the former technique gives a more accurate statistical size analysis.^47^

**Figure 2:**
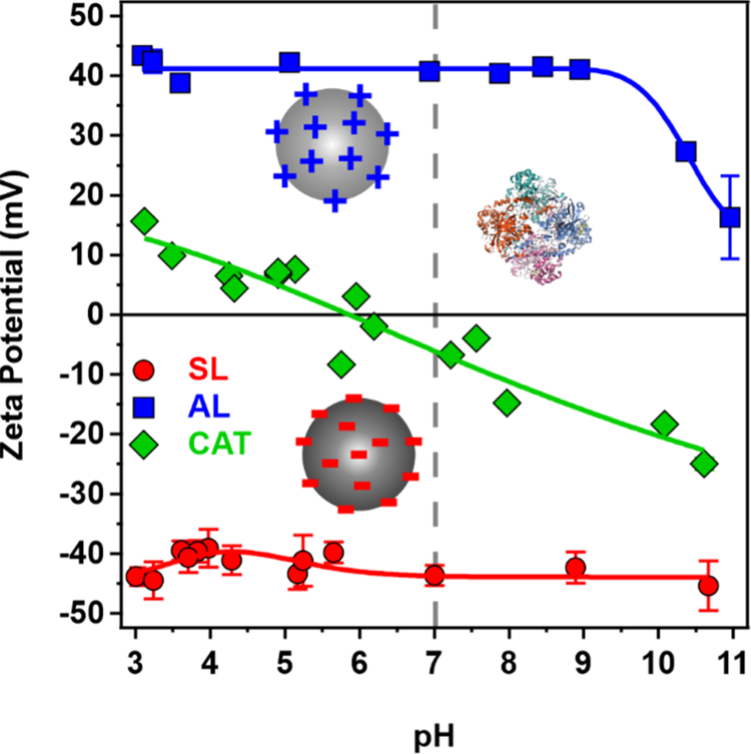
ζ-Potential values of AL and SL particles and the CAT enzyme as a function of pH. The solid lines serve to guide the eye, while the dashed lines indicate the pH 7 charge situation.

The ζ-potentials of the particles and the CAT enzyme were measured by electrophoretic light scattering as a function of pH (Figure 2).

The ζ-potential of the SL particles was around −40 mV over the entire pH range studied. The AL particles show a similar magnitude in the ζ-potential values between pH 3 and 9; however, at higher pH, they decreased significantly due to the deprotonation of the surface amidine groups. The data for the CAT enzyme indicate a slightly positive charge at low pH; however, with an increase in the pH, the ζ-potential values decrease, and the neutral state of CAT, the so-called isoelectric point (IEP), is located around pH 5.8, which is in good agreement with the literature data.^57^ Beyond the IEP, the ζ-potential of the enzyme continuously decreases, confirming the negative CAT charge under such conditions. This behavior is due to the protonation equilibria of the amino acid side chain groups in the protein chain.

### Coating of SL Nanobeads with GC and MGC Polyelectrolytes

**Figure 3:**
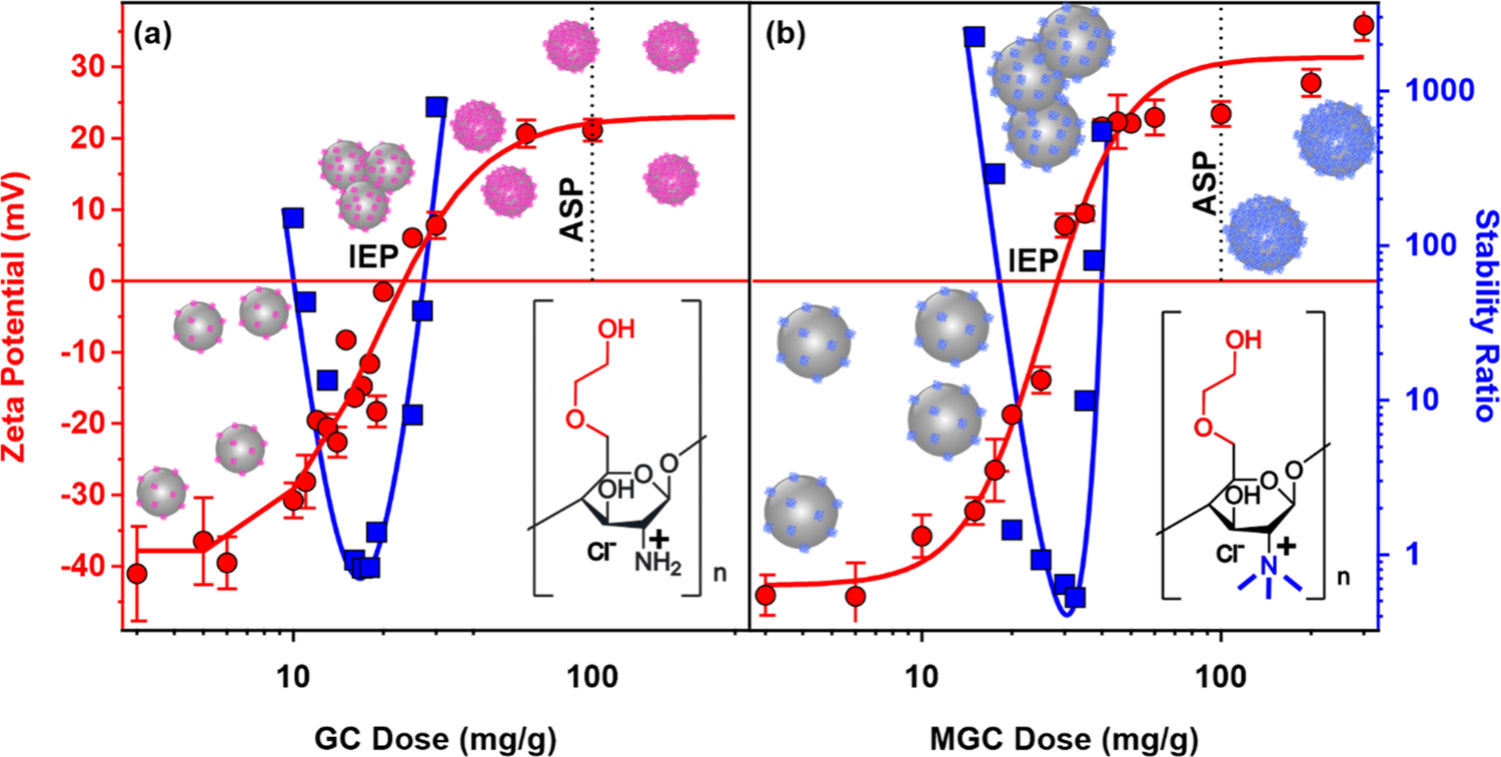
ζ-Potential (red circles, left axis) and stability ratio (blue squares, right axis) data of the SL particles as a function of GC (a) and MGC (b) doses, with the structure of the water-soluble chitosan derivatives in the insets. The measurements were carried out at pH 7 and 1 mM ionic strength adjusted with NaCl (mg/g refers to mg of the polyelectrolyte per 1 g of the particles). The solid lines serve as a guide to the eye.

Positively charged chitosan derivatives are expected to adsorb strongly on oppositely charged particles through electrostatic interactions.^30^ Therefore, SL nanobeads were coated with GC and MGC polyelectrolytes, the doses of which were optimized to achieve high colloidal stability and a positively charged surface for CAT immobilization in the further steps. Electrophoretic mobilities were first determined and converted to ζ-potentials using eq 5 to probe the influence of GC and MGC adsorption at different doses on the charging behavior of the SL (Figure 3).

Overall, the charge characteristics of the systems were very similar in the presence of both polymers. Accordingly, at doses of up to 6 mg/g, the polyelectrolytes do not significantly affect the original ζ-potential of the SL particles, which was around −40 mV. A further increase in the polyelectrolyte dose led to an increase of the ζ-potential values, which clearly indicates the adsorption of polyelectrolytes on the surface. Such an adsorption process resulted in charge neutralization at the IEP, which is located at 24 and 28 mg/g for the GC (Figure 3a) and MGC (Figure 3b), respectively. Note that this IEP, in contrast to the IEP of bare CAT discussed above, is due to charge neutralization upon the adsorption of oppositely charged polyelectrolytes on SL. Above the IEP, charge reversal occurred, and the adsorption continued until the ζ-potentials reached the adsorption saturation plateau. This onset (ASP) was determined to be 100 mg/g for both polyelectrolytes. Note that the accuracy of the methodology for obtaining the ASP value was about 15%. A similar charge reversal was reported at solid–water interfaces,^35,58−61^ but this is the first report on systems containing oppositely charged water-soluble chitosan derivatives and nanoparticles. The driving force behind such a charge reversal process includes intermolecular interactions, entropy effect, and charge correlation.^62^ These tendencies in the potential indicate that the SL nanobeads are fully coated with GC or MGC at the ASP, and further added polyelectrolytes remain dissolved in the bulk solution.

The stability ratio values were determined by time-resolved DLS measurements (Figure S3a,b for GC and MGC, respectively) as a function of GC (Figure 3a) or MGC (Figure 3b) doses. At low coverage, where the particles possess mainly the original charge of the SL, they form stable dispersions due to the repulsive electrostatic force provided by the ionized surface sulfate groups. With increasing polyelectrolyte dose, the stability ratio values reach a minimum near the IEP in both cases. Around this point, the overall charge of the particles is close to zero, the attractive van der Waals interactions come to the fore, and every collision of the particles results in dimer formation. These are the so-called diffusion-controlled conditions if only electrostatic interparticle forces are present. A further increase in the polyelectrolyte doses resulted in an increase of the stability ratio. Around the ASP, the positive charges of the adsorbed polyelectrolyte provide strong electrostatic repulsive forces between the particles giving rise to stable dispersions of high or not even measurable stability ratio data. Such a relationship between the charge-aggregation processes is in line with the Derjaguin, Landau, Verwey, and Overbeek (DLVO) theory, which indicates that the major interparticle forces are the repulsive double layer and attractive van der Waals forces.^47,49,63^ However, other types of forces reported earlier across polymer-coated surfaces (e.g., steric, bridging or depletion interactions)^64^ may also be present, which is indicated by stability ratios lower than unity (Figure 3b), but their extent is smaller than that of DLVO origin.

The above findings confirm that at a 100 mg/g polyelectrolyte dose, the SL-GC and SL-MGC particles are positively charged and form highly stable colloids, which may serve as promising carriers for subsequent CAT immobilization, as discussed later.

### Colloidal Stability in Salt Solutions

**Figure 4:**
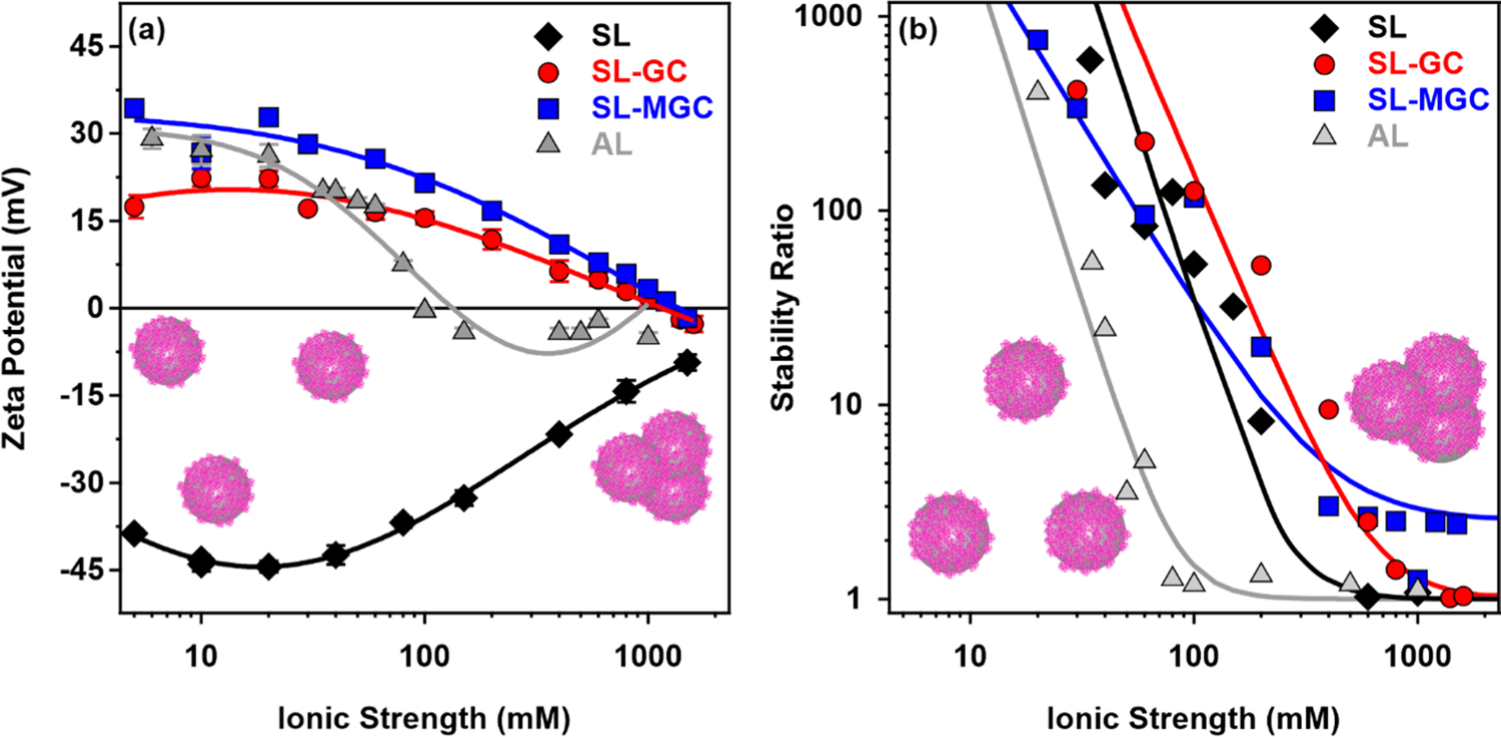
ζ-Potentials (a) and stability ratios (b) of AL, SL, SL-GC, and SL-MGC particles as a function of ionic strength adjusted with NaCl. The measurements were carried out at pH 7 at a particle concentration of 6 mg/L. The solid lines are a guide to the eye in (a) and fit with eq 3 in (b).

Ionic strength is an important factor in the preparation, stability, and applications of biocatalytic systems.^65−67^ Moreover, assessing the resistance against salt-induced aggregation is a useful tool to compare the colloidal stability of different particle systems and to reveal the possible interparticle forces.^68^ Therefore, the charging and aggregation features of the obtained particles were tested over a wide range of salt concentrations. The ionic strength was systematically changed in the dispersions of the bare and polyelectrolyte-functionalized particle systems. The ζ-potentials (Figure 4a) and stability ratios (Figure 4b) were determined and the trends in the data obtained were compared.

By increasing the ionic strength, the magnitude of the ζ-potentials decreased in all systems owing to the screening effect of the dissolved salt constituents^49,50,68^ on the surface charges (Figure 4a). Note that there is a considerable difference in the charging behavior in the case of bare particle systems. The SL remained negatively charged over the entire salt concentration range but the ζ-potential of the bare AL particles reached zero at around 100 mM and became slightly positive at higher salt concentrations, indicating the adsorption of chloride ions on the AL surface along with charge screening. The trends in the potentials of the functionalized systems are nearly the same; however, the SL-MGC particles possess higher ζ-potentials over the entire salt concentration range studied. Besides, weak charge reversal of SL-GC and SL-MGC was observed at around 1000 mM. This was again due to the specific adsorption of chloride ions on the positively charged surfaces.

The aggregation behavior at different electrolyte levels was investigated in the bare (Figure S4a,b for AL and SL, respectively) and functionalized (Figure S5a,b for GC and MGC, respectively) particle systems. The obtained stability ratios are shown in Figure 4b, while the critical coagulation concentration (CCC) was determined using eqs 3 and 4 by fitting the stability ratio data. In brief, CCC is the salt concentration (equal to the ionic strength in monovalent electrolyte solutions) that separates the slow and fast particle aggregation regions. This is a useful measure to quantify the sensitivity of colloidal or nanoparticles to salt-induced aggregation.^68^ Accordingly, high stability ratios indicate stable dispersions at low salt concentrations, while they decrease with increasing ionic strength and become unity above the CCC. The tendency in the data followed the prediction of the DLVO theory; however, some discrepancies were observed for SL-MGC at high ionic strengths. In this system, the stability ratio did not reach 1, indicating the presence of additional, non-DLVO, repulsive forces leading to lower aggregation rates and, thus, higher stability ratios. It was assumed that this force originates from the steric repulsion developed by overlapping polyelectrolyte chains upon the approach of the SL-MGC particles. This phenomenon was reported earlier for particle–polyelectrolyte systems, especially at high salt levels, where the adsorbed chains form an extended configuration on the surface giving rise to thicker polyelectrolyte layers and subsequently, to steric repulsion.^64,69^ Such an additional force is most likely responsible for the different slopes in the slow aggregation regime for the SL-MGC particles.

As expected from the charge density data (Table S1), the bare SL particles possessed a significantly higher CCC value due to the higher magnitude of the charge density compared to the bare AL (Table S1). The functionalization of the SL particles with the water-soluble chitosan derivatives resulted in a remarkable increase in the CCC values, which clearly indicates the improved colloidal stability of the SL particles upon functionalization with GC and MGC. Similar results were reported earlier for the polyelectrolyte-coated particle dispersions,^46,50,70^ but no data are available on the stabilization effect of water-soluble chitosan polyelectrolytes on nanobeads.

Note that the charge density data (Table S1) at the slip plane calculated using the Grahame equation (eq 6) indicate weaker repulsive double-layer forces for the SL-GC and SL-MGC particles than for SL. Nevertheless, the trend in CCC was the opposite. This suggests the presence of non-DLVO repulsive forces in the functionalized particle systems, which are likely attributable to additional steric forces, as discussed above. Moreover, the fast aggregation rates calculated beyond the CCC values were found to be very similar for the bare SL, SL-GC, and AL particles, while they were significantly smaller for the SL-MGC particles, which again indicates the presence of steric repulsion between the polyelectrolyte-coated nanobeads.

### Immobilization of CAT

**Figure 5:**
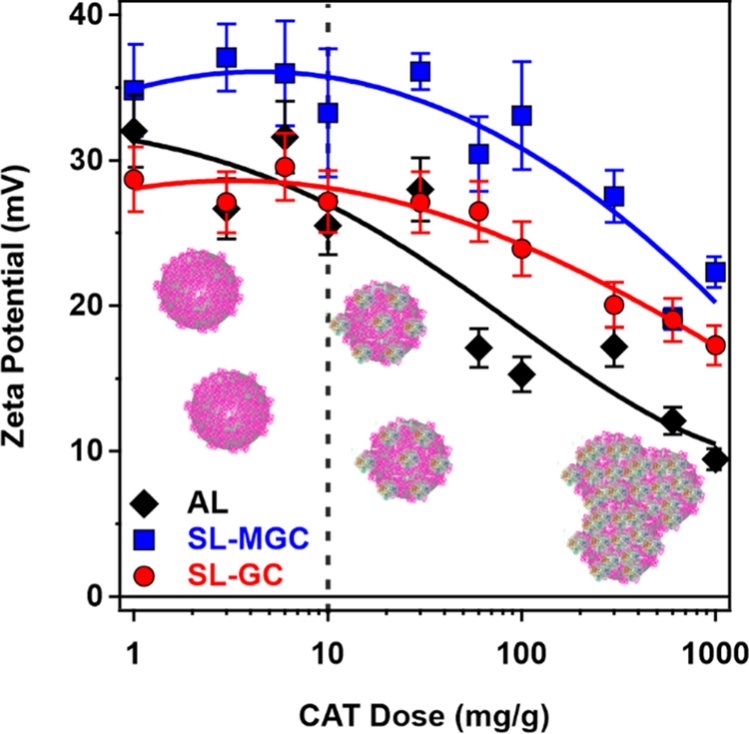
ζ-Potentials of the AL, SL-GC, and SL-MGC nanobeads as a function of the CAT dose (mg of CAT per gram of particle) at a particle concentration of 6 mg/L and ionic strength of 1 mM at pH 7. The polyelectrolyte dose was 100 mg/g for both SL-GC and SL-MGC. The solid lines are a guide to the eye.

As shown in Figure 2, CAT has a net negative charge at pH 7; therefore, it is expected to adsorb on the positively charged AL, SL-GC, and SL-MGC by electrostatic attraction. However, hydrophobic interactions and hydrogen bonding may also play a role in the adsorption process.^30,36,37^ It should be noted that extensive enzyme adsorption may affect the charging and aggregation properties of carrier particle systems. It is important to avoid a significant decrease in the magnitude of the surface charge upon enzyme immobilization because it may lead to the weakening of the double-layer repulsion and particle aggregation. To explore such a charging behavior, the influence of CAT adsorption on the ζ-potential values of the carrier particles was probed at different CAT concentrations (Figure 5).

The adsorption of CAT is indicated by the decrease of the ζ-potential values with increasing protein dose. Slight differences were observed in the tendencies, and the decrease in the potential was more pronounced for the AL system. Although the trends for the functionalized (SL-GC and SL-MGC) systems were similar, somewhat higher ζ-potential values were observed for the SL-MGC system over the entire CAT dose regime studied. These results revealed that for an enzyme dose lower than 10 mg/g, the charge features of the different systems were not affected by the amount of CAT added, indicating that the colloidal stability of the dispersed systems was preserved upon enzyme immobilization. Beyond this loading, the potential decreases, which may lead to unwanted particle aggregation as explained above. Considering these results and our previous experience with antioxidant enzyme immobilization,^30,35,70^ 10 mg/g CAT was selected for the composite nanoparticles (denoted as AL-CAT, SL-GC-CAT, and SL-MGC-CAT).

**Figure 6:**
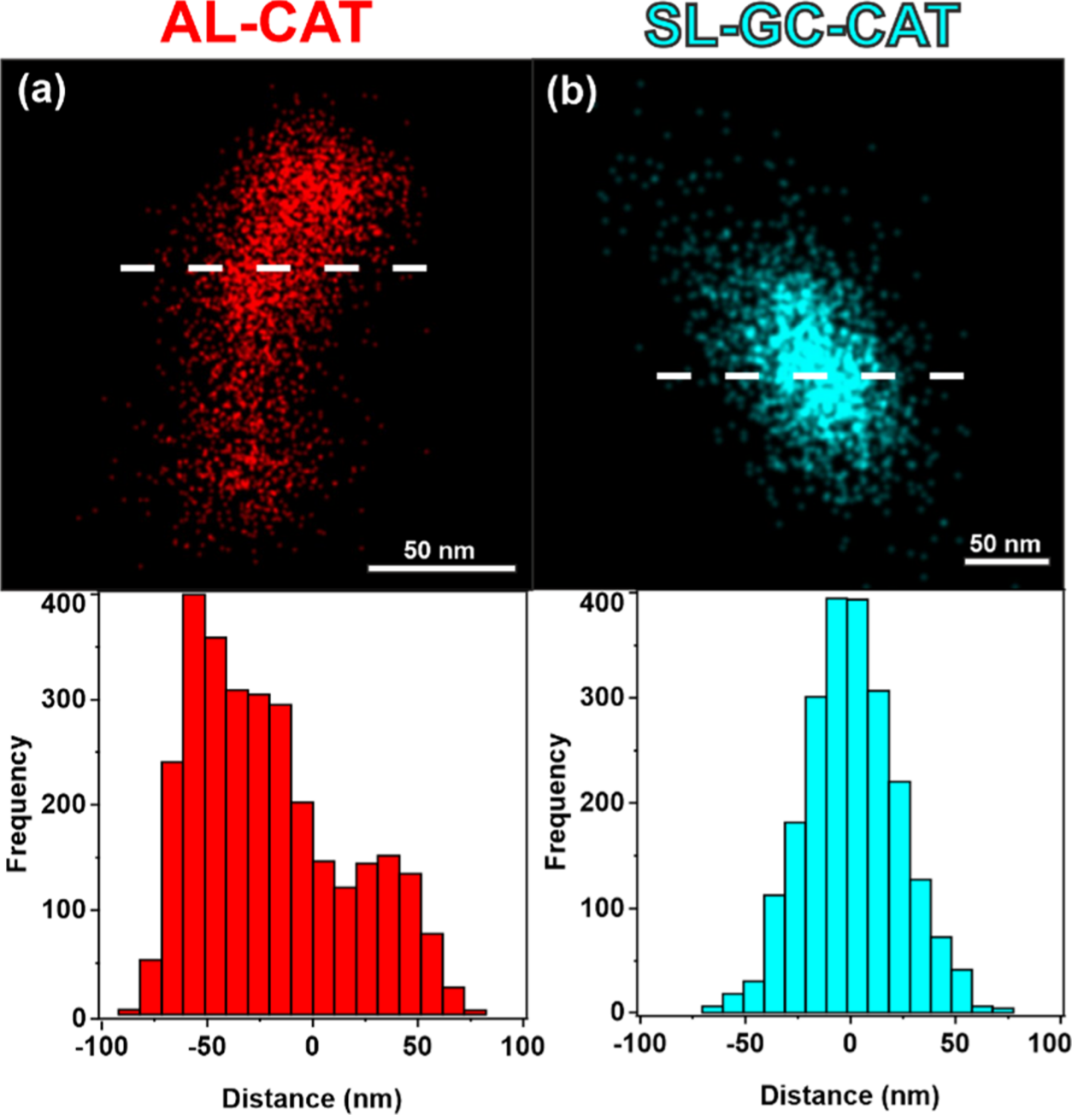
Top: Transmitted light dSTORM image of the focal points of the AL-CAT (a) and SL-GC-CAT (b) composite materials. Bottom: Blinking events of the fluorophores are plotted as a function of distance along the white dashed line shown in the top figures.

The immobilization of CAT on the oppositely charged AL and SL-GC was confirmed by the dSTORM technique (Figure 6a,b for AL-CAT and SL-GC-CAT, respectively).

The transmitted light image shows several well-concentrated foci in the distant red range of light, indicating the presence of CAT in the composite particles. After data acquisition, dSTORM revealed the spatial extent of the blinking events and blinking frequency of the fluorophores as a function of distance along the white dashed line in Figure 6. The dimensions of the flashes were about 100 nm, which is in good agreement with the diameters of the AL and SL nanoparticles. These results provide unambiguous evidence of the adsorption of CAT on nanoparticulate AL and SL-GC supports.

### Trend in CAT Activity upon Immobilization

The enzymatic activities of the native and immobilized CAT were tested using a standard spectrophotometric assay,^56^ in which the activities were determined from the remaining H_2_O_2_ concentration after the reaction was terminated. For better comparison, the enzyme concentration was kept constant in the immobilized and bare systems. The enzymatic activities of the composite materials were also determined at different time points to explore the initial, short, and long-term activities, i.e., to probe storage stability and reusability.

**Figure 7:**
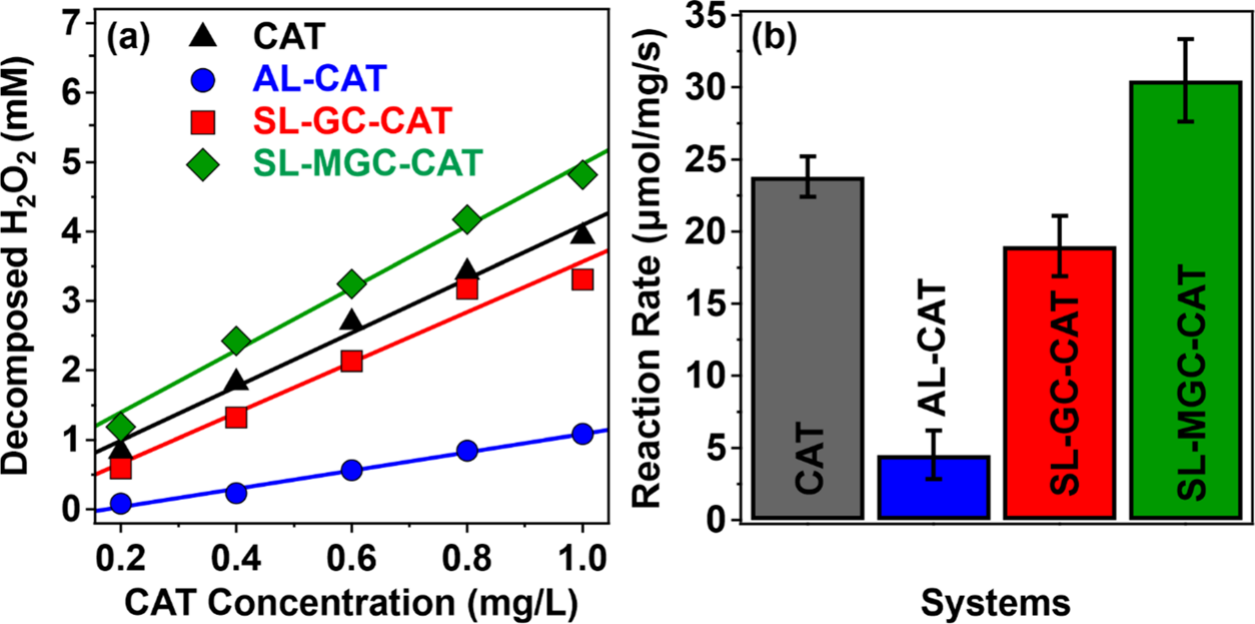
(a) Amount of decomposed H_2_O_2_ after 180 s of reaction time as a function of CAT concentration. The initial substrate concentration was 6.7 mM. (b) Reaction rate data normalized to the CAT concentration and reaction time.

First, the amount of decomposed H_2_O_2_ was determined at different CAT concentrations in the native and immobilized systems. As shown in Figure 7a, CAT immobilized on the AL support possessed significantly lower enzymatic activity than the other systems and it decomposed only 16% of the substrate even at higher CAT concentrations within the time studied.

For the freshly prepared samples, CAT immobilized on the water-soluble chitosan derivatives had similar activities as the native enzyme, and the best-performing system was SL-MGC-CAT. This was also confirmed when the reaction rate of the H_2_O_2_ decomposition data was plotted for different particles (Figure 7b). The activities of the composite systems followed the order AL-CAT < SL-GC-CAT < SL-MGC-CAT. Note that the hydrophilicity of the carrier particles is expected to increase in the same sequence. The surface of AL is the most hydrophobic among the support particles, despite the presence of amidine groups on the surface. The water-soluble chitosan derivatives contain substituted glycol and methyl glycol hydrophilic groups in the polyelectrolyte chain and upon adsorption on SL, the latex surface becomes more hydrophilic. It is obvious from the results of enzymatic activity that the hydrophilic nature of the surface led to higher activity, most likely owing to the maintenance of the original conformation of the CAT enzyme on the surface of the functionalized SL nanobeads.

**Figure 8:**
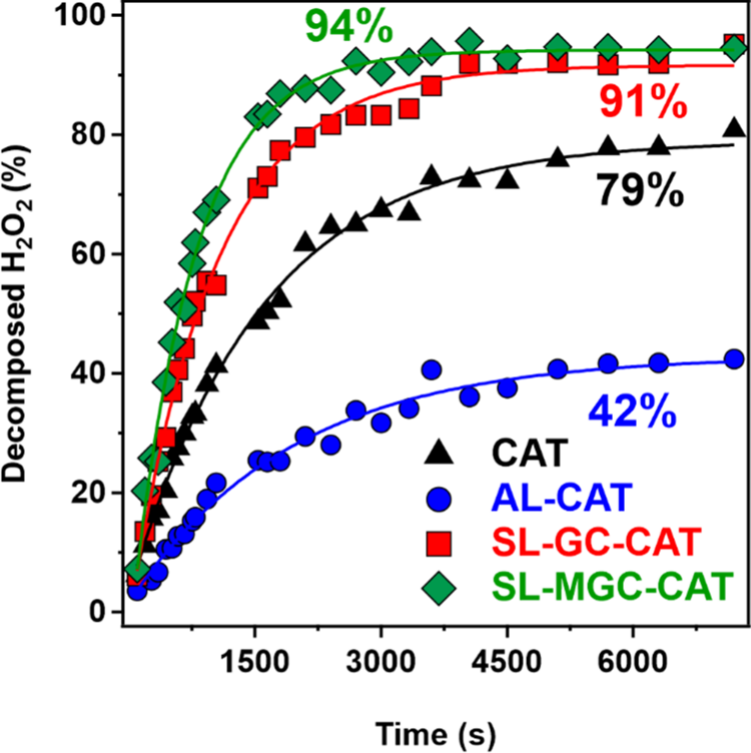
Decomposed H_2_O_2_ (in %) as a function of time. The initial substrate concentration was 6.7 mM with 0.2 mg/L CAT. The solid lines are a guide to the eye.

In the next step, the short-term stability was determined for the native and immobilized enzyme systems (see measurement details in the Experimental Section). During the 2 h test period, the amount of substrate that decomposed was determined. The AL-CAT system exhibited the lowest decomposition rate in the linear range of the data, and it could only decompose 42% of the substrate after 2 h. The native enzyme exhibited a higher initial rate; however, the final decomposition was 79%. The highest activities were observed for the polyelectrolyte-functionalized system since these composites were able to decompose nearly all of the H_2_O_2_ substrate present in the probe reaction (Figure 8). Such increased activity upon immobilization of CAT has also been observed in other systems,^30^ and can be explained by the attraction of the substrate molecules by the adsorbed polyelectrolyte chains located close to CAT on the surface. Nevertheless, no direct experimental evidence could be obtained based on the present results.

**Figure 9:**
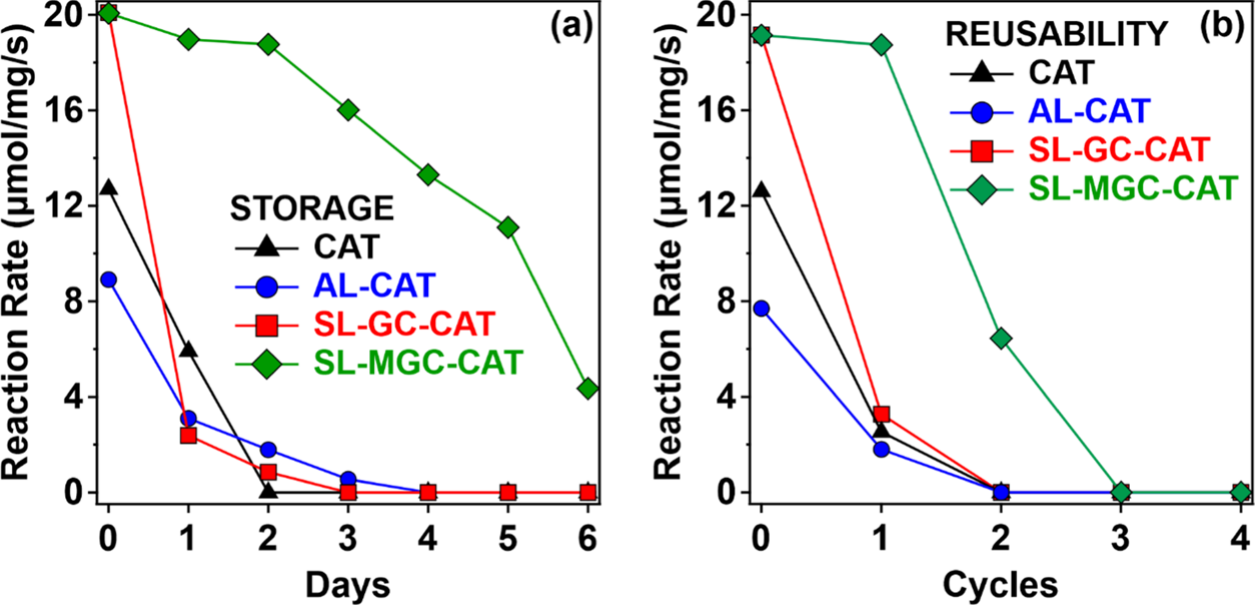
(a) Storage stability and (b) reusability of native CAT and composite particles. CAT concentrations were 10 and 0.8 mg/L in (a, b), respectively. The solid lines are a guide to the eye.

Finally, the storability and durability of the biocatalytic systems were tested. In the storage tests, samples were prepared with a CAT concentration of 4 mg/L, aliquots were taken, and the activity was tested each day for a 6-day period. The samples were stored at room temperature between the measurements. The enzymatic activities of the native and functionalized enzymes are shown as the reaction rate in Figure 9a. It was found that after the first day, the native CAT enzyme had lost its activity. Besides, no H_2_O_2_ decomposition was detected for the AL-CAT and SL-GC-CAT systems after 3 days. On the other hand, there was no significant loss in the activity of SL-MGC-CAT after 2 days, while a continuous decrease was observed thereafter with still more than 50% relative activity after 5 days, and detectable activity after 6 days. These results indicate that the storage stability of CAT can be significantly improved by immobilization on appropriately functionalized nanoparticles.

In the reusability tests (Figure 9b), the methodology closely resembled that of storage tests. However, a key difference is the repeated (daily) exposure of the stock solution to the substrate (cycles). In these experiments, the substrate was added at a higher concentration (500 mM) to avoid a significant change in the concentration of the native or immobilized CAT present in the stock solution used in the decomposition of H_2_O_2_. Despite this, the reaction rate was consistently adjusted based on the calculated enzyme concentration to maintain accuracy. It was found that the CAT, AL-CAT, and SL-GC-CAT systems significantly lost most of their activities after the first cycle. In contrast, the SL-MGC-CAT system preserved the same activity after the first cycle, while 40% of the substrate decomposing ability was measured after the third cycle.

These findings provide important insights into the optimization of enzymatic activities during immobilization, especially considering the challenging storage conditions, such as concentration, temperature, and absence of buffering agents, and these parameters can be further considered for specific applications. However, based on the present results, one can note that the colloidal approach used for the immobilization of CAT on nanoparticles with the help of polyelectrolytes is an excellent tool to achieve remarkable dispersion, structural and storage stability.

## Conclusions

In this study, polymeric AL and SL nanobeads were successfully prepared via surfactant- and emulsifier-free emulsion polymerization, demonstrating regular spherical shapes and monodisperse size distribution. The surface functionalization of SL with oppositely charged water-soluble chitosan derivatives induced charge neutralization and subsequent charge reversal, leading to highly stable colloidal dispersions of SL-GC and SL-MGC. The DLVO theory fairly explained the origin of the observed interparticle interactions, while steric repulsion was also assumed in the SL-MGC system. CAT immobilization on oppositely charged particles was confirmed by electrophoresis and microscopy. Enhanced colloidal stability and catalytic activity were observed for the polyelectrolyte-functionalized support particles, with a nearly complete decomposition of H_2_O_2_ within a reasonable time frame by the SL-GC-CAT and SL-MGC-CAT composites. Regarding storage stability and reusability, the SL-MGC-CAT system displayed remarkable stability of CAT function since significant activities were detected after 5 days and 3 cycles, respectively. These findings highlight the potential of polyelectrolyte-functionalized nanoparticles as promising carriers for enzyme immobilization, offering insights into their stability, catalytic efficiency, and possible applications in various fields where H_2_O_2_ elimination is an important task. Further optimization of the preparation conditions of the carrier nanobeads may extend their storage stability and enhance their reusability, paving the way for future advancements in enzyme adsorption onto nanoparticle supports.

## Supporting Information Available

Calibration curve for CAT assay; TEM images of AL and SL particles; hydrodynamic radius data; charge density, CCC, and aggregation rate values (PDF)
