# Supplementary material for: Optimization of Interfacial Properties Improved the Stability and Activity of the Catalase Enzyme Immobilized on Plastic Nanobeads
Source: Langmuir. 2024 Jul 27;40(31):16338–48. doi: 10.1021/acs.langmuir.4c01508 (PMC11308775; doi:10.1021/acs.langmuir.4c01508)
Supplement: Supplementary file 1 — la4c01508_si_001.pdf [file la4c01508_si_001.pdf]

Supporting Information (SI)

Optimization of Interfacial Properties Improved the Stability and Activity of Catalase Enzyme Immobilized on Plastic Nanobeads

Szilárd Sáringer,[‡] *Gergő Terjéki,*[‡] *Árpád Varga,*[ǁ] *József Maléth*[ǁ] *and István Szilágyi*[‡,*]

[‡]MTA-SZTE Lendület Biocolloids Research Group, Interdisciplinary Excellence Center, Department of Physical Chemistry and Materials Science, University of Szeged, H-6720 Szeged, Hungary

[ǁ]MTA-SZTE Lendület Epithelial Cell Signaling and Secretion Research Group, Interdisciplinary Excellence Centre, University of Szeged, H-6720 Szeged, Hungary

*Corresponding author. Email: szistvan@chem.u-szeged.hu

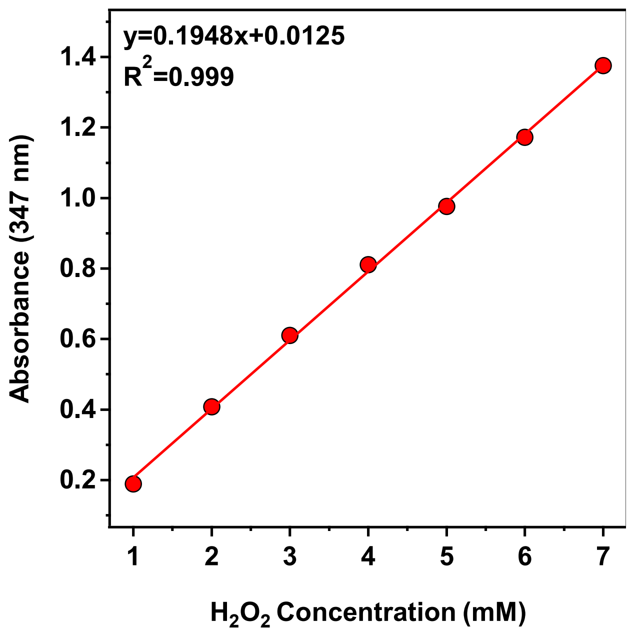

Figure S1. Absorbance versus H_2O_2 concentration data measured in the presence of ammonium molybdate at 346 nm wavelength. The parameters of the linear regression used to calculate the H_2O_2 concentrations are indicated.

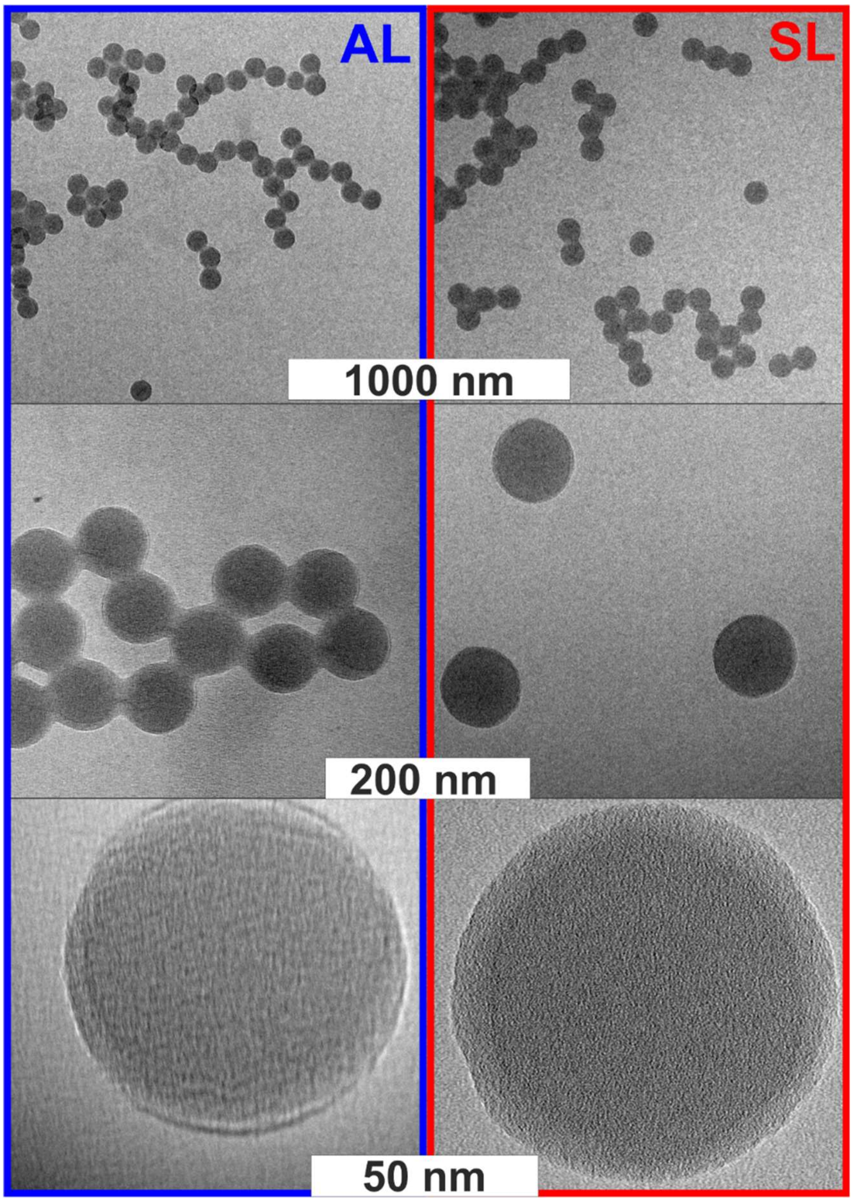

Figure S2. Bright field TEM images of the AL (left column) and SL particles (right column) at different magnifications. The images were recorded after drying the dispersions.

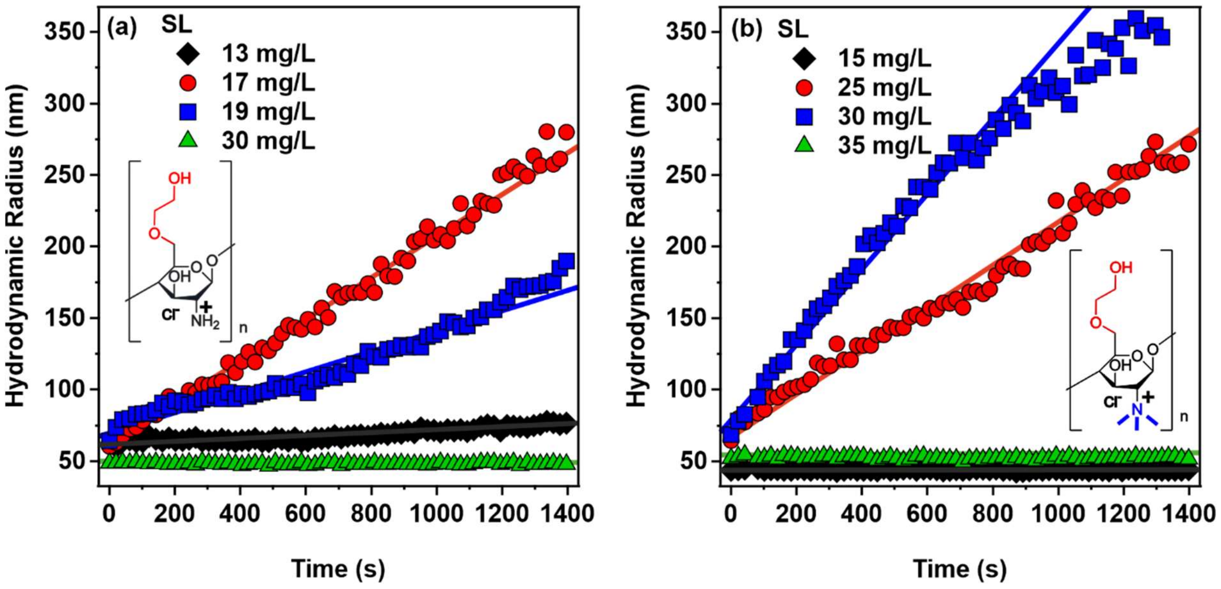

Figure S3. Representative hydrodynamic radius versus time data at constant SL concentration and different GC (a) and MGC (b) concentrations before, around and above the IEP. The linear fits were used to calculate the aggregation rate coefficients (Eq 1).

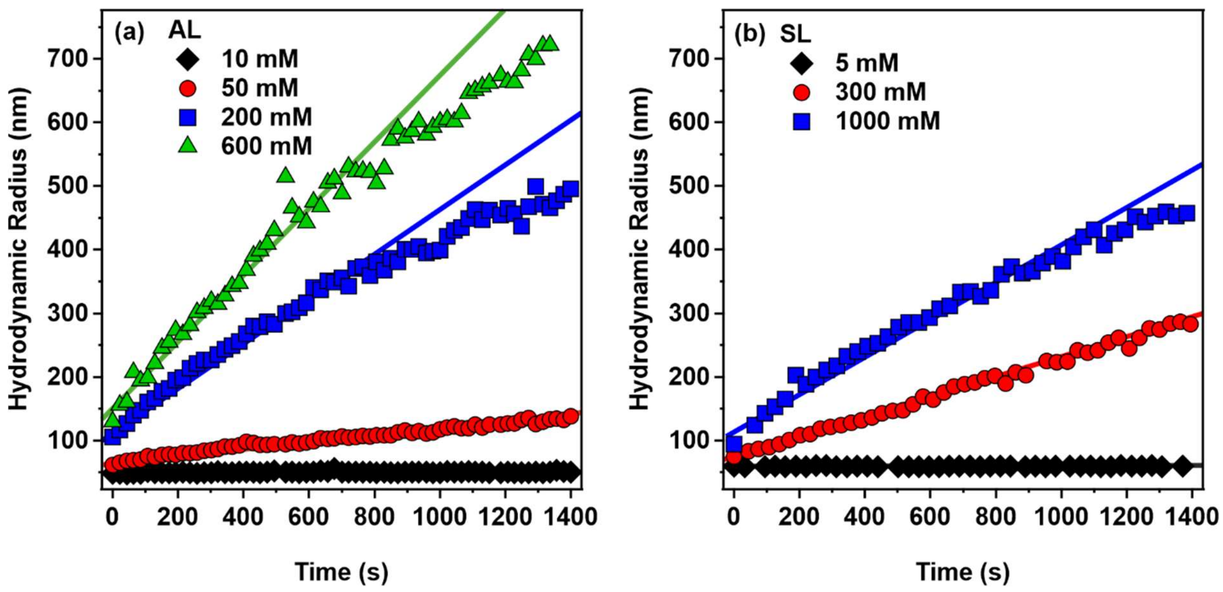

Figure S4. Representation of time-resolved DLS measurements. Aggregation of bare AL (a) and SL (b) at different ionic strength at 6 mg/L particle concentration and 1 mM ionic strength. The linear fits were used to calculate the aggregation rate coefficients (Eq 1).

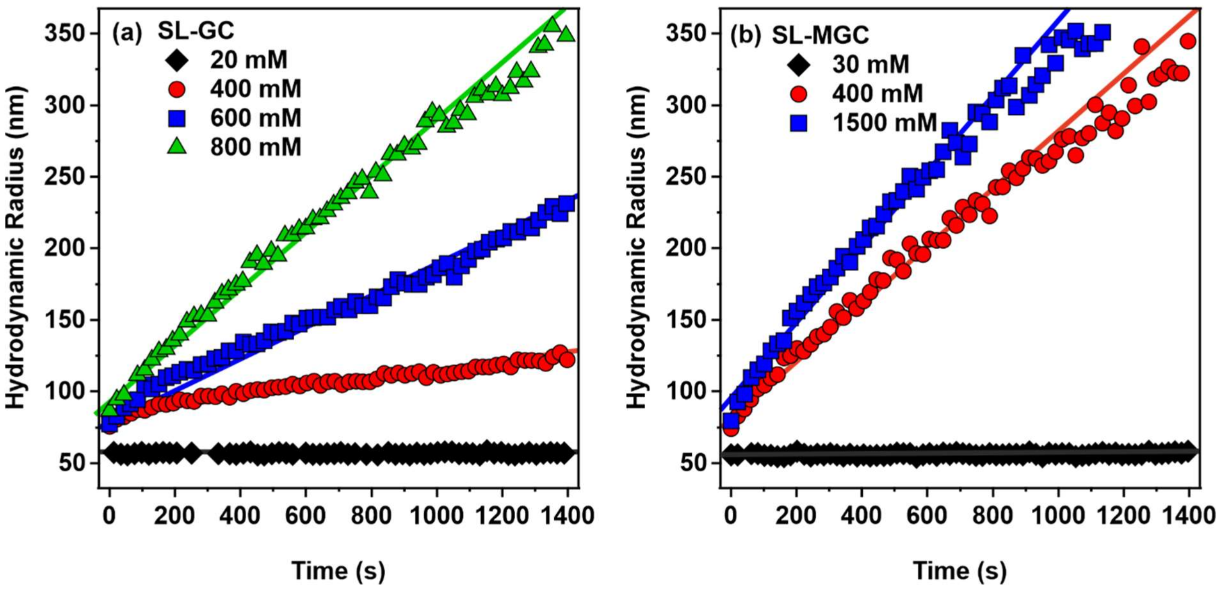

Figure S5. Illustration of time-resolved DLS measurements. Hydrodynamic radii of SL-GC (a) and SL-MGC (b) versus time at different ionic strength at 6 mg/L particle concentration and 1 mM ionic strength. The dose of both polyelectrolytes was 10 mg/g (relative to the mass of particle). The linear fits were used to calculate the aggregation rate coefficients (Eq 1).

Table S1. Charge density, CCC and fast aggregation rate of bare and composite particles.

	AL	SL	SL-GC	SL-MGC
Surface charge density (mC/m^2)[a]	9	-27	10	15
CCC (mM)[b]	82	272	634	625
Fast aggregation rate (m^3/s)[c]	2.60×10^{-19}	2.34×10^{-19}	2.28×10^{-19}	8.90×10^{-20}

[a]The charge density at the slip plane was calculated from the zeta potential versus ionic strength data with Eq 6. [b]The CCC was determined with Eq 3 and Eq 4. [c]Fast aggregation rate coefficients were calculated with Eq 1 at ionic strengths above the CCC.
